# Decoding transcriptional regulation of microglia associated with amyloid plaques

**DOI:** 10.1002/alz70855_106751

**Published:** 2025-12-25

**Authors:** Johannes C.M. Schlachetzki, Yi Zhou, Nathan Spann, Kilian Leon Kleemann, Roisin M. McManus, Michael T. Heneka, Oleg Butovsky, Christopher K. Glass

**Affiliations:** ^1^ University of California, San Diego, San Diego, CA, USA; ^2^ UCSD, La Jolla, CA, USA; ^3^ Brigham and Women's Hospital, Harvard Medical School, Boston, MA, USA; ^4^ German Center for Neurodegenerative Diseases (DZNE), Bonn, NRW, Germany; ^5^ Luxembourg Centre for Systems Biomedicine (LCSB), University of Luxembourg, Luxembourg, Luxembourg

## Abstract

**Background:**

Beyond the progressive accumulation of amyloid‐beta and hyperphosphorylated tau, Alzheimer's disease (AD) is accompanied by phenotypic changes in microglia, the innate immune cells of the brain. In particular, microglia in the vicinity of Amyloid‐plaques, also known as MGnD or DAM, are defined by distinct changes in their gene expression signature, e.g., upregulation of *Trem2* mRNA. However, the precise transcriptional mechanism that drives microglia phenotype in response to amyloid is not defined.

**Method:**

Here, we isolated microglia from the APP/PS1 transgenic mouse model followed by ATAC‐seq.

**Result:**

We provide evidence for a model in which differential activation of a common set of transcriptional regulators that includes members of the MITF/TFE, AP‐1 and EGR transcription factor families drives the amyloid‐plaque associated microglia phenotype.

**Conclusion:**

Collectively, these findings reveal the framework of the transcriptional circuitry that is used to establish a range of neurodegenerative pathology‐associated microglia phenotypes.

